# Mother-to-Child Transmission of Arboviruses during Breastfeeding: From Epidemiology to Cellular Mechanisms

**DOI:** 10.3390/v13071312

**Published:** 2021-07-07

**Authors:** Sophie Desgraupes, Mathieu Hubert, Antoine Gessain, Pierre-Emmanuel Ceccaldi, Aurore Vidy

**Affiliations:** 1 Unité Épidémiologie et Physiopathologie des Virus Oncogènes, Département Virologie, Institut Pasteur, 75015 Paris, France; mathieu.hubert@pasteur.fr (M.H.); antoine.gessain@pasteur.fr (A.G.); pierre-emmanuel.ceccaldi@pasteur.fr (P.-E.C.); 2 Université de Paris, 75013 Paris, France; 3 UMR Centre National de la Recherche Scientifique 3569, Institut Pasteur, 75015 Paris, France

**Keywords:** arboviruses, mother-to-child transmission, breastfeeding, breast milk, intestinal epithelium, mammary epithelium, Zika virus, yellow fever virus, dengue virus, Chikungunya virus, West Nile virus, barrier crossing mechanisms, viral transmission

## Abstract

Most viruses use several entry sites and modes of transmission to infect their host (parenteral, sexual, respiratory, oro-fecal, transplacental, transcutaneous, etc.). Some of them are known to be essentially transmitted via arthropod bites (mosquitoes, ticks, phlebotomes, sandflies, etc.), and are thus named arthropod-borne viruses, or arboviruses. During the last decades, several arboviruses have emerged or re-emerged in different countries in the form of notable outbreaks, resulting in a growing interest from scientific and medical communities as well as an increase in epidemiological studies. These studies have highlighted the existence of other modes of transmission. Among them, mother-to-child transmission (MTCT) during breastfeeding was highlighted for the vaccine strain of yellow fever virus (YFV) and Zika virus (ZIKV), and suggested for other arboviruses such as Chikungunya virus (CHIKV), dengue virus (DENV), and West Nile virus (WNV). In this review, we summarize all epidemiological and clinical clues that suggest the existence of breastfeeding as a neglected route for MTCT of arboviruses and we decipher some of the mechanisms that chronologically occur during MTCT via breastfeeding by focusing on ZIKV transmission process.

## 1. Introduction: Arboviruses, Breastfeeding, and Milk-Borne Viruses

In 1881, Carlos Finlay was the first to hypothesize that a virus, especially yellow fever virus (YFV), was able to be transmitted by mosquitoes [1]. As a revolution in the virology field, Finlay’s hypothesis was furthermore confirmed in 1900. Thereafter, other viruses were found to be transmitted by mosquitoes and other arthropods, such as ticks, phlebotomes, or sandflies. In this context, the World Health Organization (WHO) [2] defined in 1967 arthropod-borne viruses or “arboviruses” as viruses that are naturally maintained through biological transmission between susceptible vertebrate hosts by hematophagous arthropods. Nowadays, more than 500 arboviruses have been identified, belonging to different viral families and orders (*Flaviviridae, Togaviridae, Rhabdoviridae, Reoviridae, Bunyavirales*) but sharing the characteristic of being transmitted by arthropod vectors. About 20% of them are known to be pathogenic for humans [3].

In humans, arbovirus infections are essentially characterized by an asymptomatic carriage or the development of mild non-specific symptoms such as fever, rash, arthralgia, myalgia, or conjunctivitis [4]. More specifically, some arboviruses can also cause severe symptoms such as brain disorders (West Nile virus (WNV), tick-borne encephalitis virus (TBEV), Zika virus (ZIKV), etc.), hemorrhagic fevers (Crimea-Congo hemorrhagic fever virus (CCHFV), YFV, dengue virus (DENV), etc.) [5], and/or severe arthritis (Chikungunya virus (CHIKV), Sindbis virus (SINV), Ross River virus (RRV), etc.) [6]. Although arboviruses are transmitted via arthropod bites, other modes of transmission have been highlighted during the last ten years.

Since ZIKV infections of pregnant women were revealed to be associated with severe outcomes in fetuses (called congenital Zika syndromes (CZS)), mother-to-child transmission (MTCT) of arboviruses must be further studied. MTCT of arboviruses could occur before (antenatal or congenital), during (perinatal), and after (post-natal) delivery. While prenatal MTCT of arboviruses and underlying mechanisms are nowadays well studied, especially for ZIKV, perinatal and post-natal transmissions remain understudied. Perinatal transmission of arboviruses to newborns could occur during delivery in case of lesions that allow blood exchanges between a viremic mother and her infant. Nowadays, perinatal transmission of arboviruses during delivery is considered low but has not really been investigated.

In this review, we focus on post-natal transmission of arboviruses during breastfeeding and the associated mechanisms.

Breastfeeding is defined as a post-natal continuum of the link between the mother and the child. In opposition to the prenatal link of the mother–fetus dyad ensured by blood exchanges through the placenta, the post-natal link between the mother and her newborn(s) during breastfeeding is not systematic. This cultural practice is heterogeneously distributed across the world: according to UNICEF, 20% of children who live in high income countries never receive breast milk, against only 4% in low and middle income countries.

The WHO recommends to initiate breastfeeding within the first hour of life and keep exclusively breastfeeding during the first six months to a year of life, depending on the newborn’s and mother’s needs. Breastfeeding has been shown to provide many benefits to the newborns. Not only does it meet the infant’s nutritional needs by providing energy and nutrients, but it also promotes cognitive development and protects the newborn against infectious and chronic diseases [7]. In order to meet the changing needs of the infant as it grows, the composition of breast milk evolves between the first hours and weeks after delivery. The first milk, called colostrum, plays an essential role in the post-natal maternal immunity transfer as it is rich in blood factors such as maternal immune cells. As the colostrum’s composition in lactose and lipids increases, the milk becomes more nutritious and is first called “transitional”, then “mature” [8].

Although breast milk provides the newborn with nutritious, developmental, and protective factors, it can also transmit deleterious chemical agents from the maternal bloodstream such as nicotine [9], alcohol [10], and drugs [11], or pathogens such as bacteria or viruses [12]. Historically, the first milk-borne virus identified was the Mouse Mammary Tumor Virus (MMTV) [13]. Since then, three viruses were recognized to be efficiently transmitted from mother to child during breastfeeding in humans, resulting in a chronic infection in the newborn: cytomegalovirus (CMV), human immunodeficiency virus type 1 (HIV-1), and human T-cell leukemia virus type 1 (HTLV-1) [12,14]. Because components of other viruses were detected in the colostrum and the breast milk (infectious viral particles, viral genome, viral proteins), questions concerning the potential transmission of other viruses during breastfeeding have been raised. Several arboviruses have been detected in breast milk of infected mothers, raising alarm concerning the risk of transmission of arboviruses to newborns during breastfeeding [15,16,17,18,19,20,21,22,23,24,25].

## 2. Epidemiological and Clinical Aspects of Milk-Borne Arboviruses

Several arboviruses have been described as being excreted in breast milk. In most cases, only the viral genome was detected, but isolation of infectious particles from some samples raises important questions regarding the risk of transmission to the infant through breastfeeding. To date, the presence of four flaviviruses (WNV, DENV, YFV, ZIKV) and one alphavirus (CHIKV) have been reported in colostrum and/or human breast milk (Table 1).

**CHIKV**—After returning from a region endemic for CHIKV, DENV, and ZIKV, a 28-year-old woman developed non-specific symptoms such as rashes and headaches with asthenia [15]. Three days after the onset of symptoms, only CHIKV infection was confirmed by RT-PCR. CHIKV viral genome was also detected in breast milk 3 and 23 days after the onset of symptoms, including after viral clearance from the bloodstream, demonstrating viral persistence of CHIKV in breast milk. Breastfeeding of the 3-month-old infant was interrupted as soon as the symptoms appeared, thus avoiding infection of the baby.

**DENV**—The viral genome of DENV has been detected in breast milk of infected women from the first day to 14 days after onset of symptoms. The viral excretion kinetics of DENV in breast milk of infected women was monitored a few days before or after delivery during a dengue epidemic in New Caledonia (December 2012 to October 2013) [16]. Unlike viremia, whose representative curve over time has the appearance of a peak, the viral excretion of DENV RNA in breast milk is characterized by a plateau associated with prolonged detection of the viral genome 18 days after birth [16]. In this study, neonatal infection associated with lack of viremia in umbilical cord blood suggests potential transmission through breast milk. In another study, infectious particles of DENV were isolated from breast milk, and MTCT of DENV during breastfeeding was strongly suggested in an infant with DENV infection after being breastfed by an infected woman [18].

**WNV**—On 2 September 2002, a 40-year-old woman gave birth and received two red blood cell concentrates from a donor, later diagnosed as infected with WNV. Thirteen days later, anti-WNV-specific immunoglobulins M (IgM) were detected in the cerebrospinal fluid (CSF), confirming mother’s infection with WNV. At day 16, WNV genome was detected in her breast milk. Since breastfeeding was started on the day of birth, molecular and serological diagnosis tests were performed on the infant, and no viral RNA was detected but WNV-specific IgM were present [19]. The viral genome of WNV was also detected in the colostrum of other women who had been infected before delivery [20].

**YFV**—Following vaccination against yellow fever, three cases of MTCT of the YFV 17DD vaccine strain via breastfeeding were suspected [22], of which one case was confirmed [21]. On 7 April 2009, a 22-year-old woman who gave birth by caesarean section went to the hospital for post-surgical intervention and received the vaccine strain 17DD of YFV because of the endemic context of yellow fever in Brazil. From 15 April, 23 days after delivery, the breastfed infant developed fever accompanied by increasingly frequent and intense convulsions. On 19 April, viral RNA from the 17DD strain as well as YFV-specific IgM were detected in the cerebrospinal fluid (CSF) of the infant. Although the breast milk was not tested, transmission of the YFV vaccine strain 17DD by breastfeeding was confirmed following sequencing of the isolate from the infant’s CSF. Although the presence of the 17DD vaccine strain was not investigated in breast milk, it was found that wild-type YFV is excreted in breast milk four days after the onset of symptoms [26]. The transmission of the 17DD vaccine strain to infants through breastfeeding was accompanied by severe neurological pathogenicity, forcing authorities to contraindicate vaccination against yellow fever during breastfeeding.

**ZIKV**—Due to the amplitude of recent ZIKV outbreaks, especially in Latin America in 2015–2016, the WHO declared a public health emergency of international concern (PHEIC) and more studies were conducted on ZIKV. The viral genome of ZIKV has been detected in the breast milk of several infected women [17,22,23,25,27,28,29,30,31,32]. This was quantified in several studies, revealing substantial viral loads (2.9.10^4^ to 2.4.10^6^ copies/mL) [17,25,27], significantly higher than those detected in the blood circulation (0 to 7.0.10^4^ copies/mL) [22]. Large viral loads were mostly detected in breast milk between 3 and 5 days after the onset of symptoms, however they could also persist far longer [17,30] and even after viral clearance from the blood stream [17,27]. Thus, Sotelo et al. found a viral excretion of ZIKV in breast milk 14 days (2.4.10^6^ copies/mL), 23 days, and more than 30 days (2.2.10^5^ copies/mL) after the onset of symptoms, demonstrating the ability of ZIKV to persist in this biological fluid and to be excreted during lactation [17]. The presence of infectious particles in breast milk has also been repeatedly demonstrated in both colostrum and breast milk [17,23,25,28] but, to our knowledge, the viral titer was never determined. Among the studies reporting the presence of viral genome and/or infectious particles in breast milk, four cases of probable transmission of ZIKV from mother to infant through breast milk have been found [23,27,31]. The newborns’ infections were demonstrated either by positive RT-qPCR in serum and urine/saliva samples, or by the development of secondary microcephaly (post-natal microcephaly) by one infant. Although vector-borne transmission can never be excluded, one case [24] of ZIKV transmission to a newborn strongly suggests transmission during breastfeeding as ZIKV vRNAs extracted from breast milk of the infected mother and urine of the 5-month-old infant showed sequence identity to 99% [23,24]. In addition, the advanced age of the infant (5 months) and the fact that he was sheltered from potential vectors (air-conditioned housing) strengthen the plausibility of this mode of transmission. Viral transmission during breastfeeding could therefore have been involved in the increase of infected infants during the ZIKV epidemic in Latin America (2015–2016), though any quantitation is impossible due to mosquito vectors pervasiveness.

Thus, MTCT of arboviruses, although difficult to demonstrate, has so far been confirmed for YFV and strongly suggested for ZIKV. As breastfeeding is essential for the development and survival of infants in many underdeveloped countries, abstinence from this practice due to maternal viral infection can be fatal for the infant. Therefore, it is necessary to understand the molecular and cellular mechanisms involved in the transmission of these viruses during breastfeeding in order to propose appropriate therapeutic strategies while maintaining the breastfeeding process.

## 3. Mother-to-Child Transmission of Arboviruses during Breastfeeding and Underlying Mechanisms

**Figure 1:**
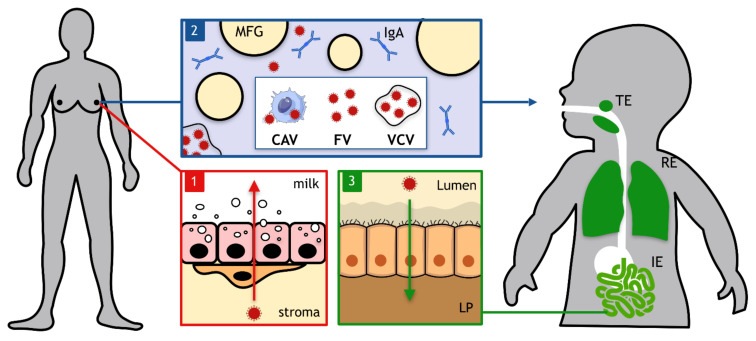
A chronology of the three main events occurring during viral mother-to-child transmission by breastfeeding. The first event consists of viral dissemination to mother’s mammary glands where viral infectious entities need to cross the mammary epithelium to be excreted in breast milk (red box). Then, viral infectious entities that could be either cell-associated viruses (CAVs), free viruses (FVs) or vesicle-cloaked viruses (VCVs) transit in breast milk in the presence of diverse components such as milk fat globules (MFGs) or immunoglobulins A (IgA). At this stage, conservation of viral infectivity needs to be ensured in breast milk (blue box). Finally, viral infectious entities can cross tonsillar (TE), respiratory (RE), and/or intestinal (IE) epithelia to infect the breastfed newborn. As an example, viral infectious entities present in the small intestine’s lumen need to cross the monostratified intestinal epithelium to reach the lamina propria (LP) (green box).

Conceptually, three main steps chronologically occur during MTCT of viruses during breastfeeding (Figure 1). First, viral tropism and/or dissemination to the mother’s mammary glands is a prerequisite for the virus to be excreted in breast milk. Then, viral particles need to cross the mammary epithelium to be delivered into the breast milk. Mechanisms involved in the productive infection of epithelial cells, their associated secretory machinery as well as the physiology of the blood–milk barrier are important elements to better understand this first step. Secondly, sufficient structural stability and persistence of viral infectivity in breast milk need to be guaranteed for a virus to be transmitted during breastfeeding. The efficiency of several treatments of contaminated breast milk (pasteurization, freezing, etc.) to decrease viral infectivity are also addressed. Third, transmissibility of milk-borne arboviruses to the breastfed newborn relies on the ability of the virus to cross anatomical epithelial barriers of the tonsillar, respiratory, and/or gastro-intestinal tracts. Identification of entry sites and facilitating factors could help to implement prevention strategies. Since MTCT of arboviruses during breastfeeding has been neglected for a long time, we emphasized this section on ZIKV, for which data were obtained in our laboratory and others.

**Step 1. Viral dissemination to mammary glands and excretion in breast milk.**

**The mammary gland structure and cell**—The presence of mammary glands is an exclusive characteristic of mammals. Unlike other organs, they develop both before and after birth and undergo numerous structural and functional changes during the life of female mammals, making them a dynamic organ. As exocrine glands, their main function is to produce and excrete breast milk [36].

**Figure 2:**
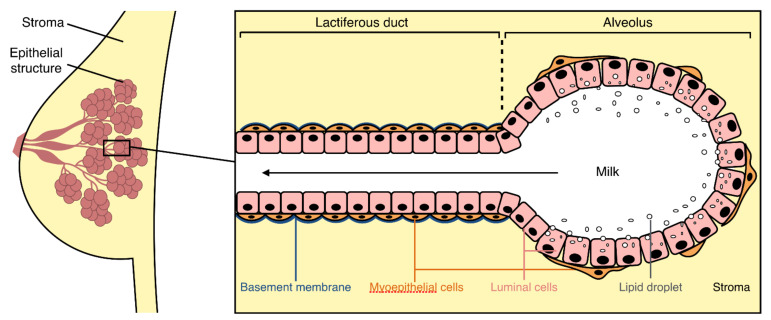
General organization of the mammary gland and its epithelium. The mammary epithelium (pink) is a ramified structure embedded in a fibro-adipose connective tissue: the mammary stroma (yellow). The epithelial structure is ramified into different levels of lactiferous ducts leading to milk-producing alveoli. The bistratified mammary epithelium (black box) is composed of luminal cells (pink) in contact with the lumen (white) and myoepithelial cells (orange) in contact with both the basement membrane (blue) and the stroma.

Like most organs, the mammary glands are made up of different cell types (stem cells, epithelial cells, fibroblasts, adipocytes, endothelial cells, immune cells, neural cells) participating in the structure and function of the organ [36]. Epithelial cells form a ramified milk-producing structure that is anchored in a mass of fibro-adipose connective tissue, the mammary stroma, which is composed of fibroblasts and adipocytes (Figure 2). Endothelial, immune, and neural cells are primarily involved in the vascular supply, lymphatic drainage, and innervation of the mammary glands, and are essential for their development and functioning.

The structure of the mammary epithelium varies between the milk ducts and the mammary alveoli, but its general composition is common to both structures (Figure 2). The mammary epithelium is composed of two cellular layers linked by intercellular junctions: an internal layer in contact with the lumen of the mammary alveoli and lactiferous ducts (luminal cells), and an outer layer resting on basal lamina in contact with the mammary stroma (myoepithelial cells) [36].

**The mammary gland as a target of viral infection**—The excretion of viral particles into breast milk requires viral spread from the bloodstream to mammary glands. Mammary alveoli are surrounded by a network of blood capillaries that allow exchanges of nutrients, oxygen, and also pathogens between the bloodstream and the mammary epithelial cells. To be excreted into breast milk, viruses need to cross a barrier (e.g., blood–milk barrier).

For HIV, mammary barrier crossing does not occur by direct infection of the epithelium as mammary epithelial cells do not express the CD4 receptor at their surface [37,38] and are not susceptible to HIV-1 infection [38]. Viral particles in breast milk could be produced by local immune cells or could have transcytosed across the mammary epithelium as endocytosed virions were observed in primary mammary epithelial cells [38].

For HTLV-1, the virus in breast milk is cell-associated [39]. Leukocytes [39] that transmigrated across the blood–milk barrier and mammary epithelial cells [40] are found infected in breast milk.

For CMV, local viral reactivation in lactating mammary glands leads to CMV shedding into breast milk [41]. Infected leukocytes also cross the blood–milk barrier as they are detected in breast milk [42].

To this day, dissemination of arboviruses to female mammary glands and subsequent excretion in breast milk are understudied. Therefore we put the emphasis on ZIKV, for which data were obtained in our laboratory [43] and by Regla-Nava et al. [44].

In our laboratory, we showed that ZIKV disseminates to the mammary glands of 129S2/SvPas- *Ifnar1^tm1Agt^* mice (A129) after intra-peritoneal or sub-cutaneous inoculation. We also demonstrated that primary human mammary epithelial cells are permissive to a productive ZIKV infection, suggesting that the human mammary epithelium could be the source for viral particles detected in the breast milk of infected mothers. As the mammary epithelium is composed of two types of epithelial cells, we assessed whether ZIKV infects myoepithelial or luminal cells. Using luminal-like and myoepithelial-like cell lines, we showed that both cell types are productively infected by ZIKV [43]. Myoepithelial cells were also shown to be infected in vivo after systemic infection of AG129 mice by ZIKV [44], confirming our results. As luminal cells specialize in the excretion of various molecules (proteins, lipids, lactose, etc.) in breast milk, their secretory mechanisms could be exploited by ZIKV for the viral shedding into breast milk. Since viral RNA has been detected in the breast milk of an infected woman up to 33 days after the onset of symptoms, it would be interesting to perform a long-term study to determine how long ZIKV is detected in mammary glands. Indeed, mammary glands could play the role of reservoir and explain the persistence of the virus in breast milk, since vRNA was absent from the woman’s bloodstream while present in breast milk.

Furthermore, our experiments were carried out on non-lactating mice so it would be interesting to study viral dissemination to mammary glands at other stages of its development (during gestation or lactation), as important structural and metabolic changes occur. The effect of lactation was studied by Regla-Nava et al. who observed high viral loads in mammary glands of lactating mice at 5 to at least 11 days after inoculation. The abundance and proliferation of breast epithelial cells during lactation could contribute to the role of reservoir of the lactating mammary glands. In addition, phenotypic changes induced by cell differentiation occurring during the lactation phase could allow the luminal alveolar epithelial cells to be more susceptible to ZIKV infection.

Finally, we observed that pups breastfed by ZIKV-infected mothers did not gain weight over time while pups breastfed by non-infected mothers grew well. In contrast, orally infected pups through intra-gastric inoculation did not show any weight loss or stagnation compared to non-infected pups. Therefore, these results suggest an effect of ZIKV infection either on the mother’s behavior regarding breastfeeding or on milk composition and/or excretion from the milk ducts. To address this hypothesis, it could be of interest to study the effect of ZIKV infection on the mammary gland architecture as well as its milk secretion and ejection functions.

**Figure 3:**
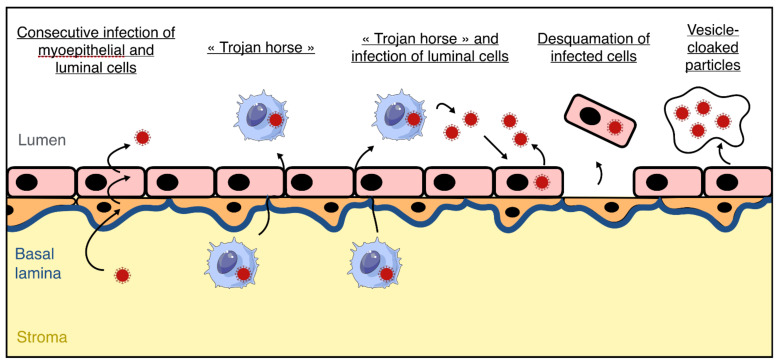
Hypothetical mechanisms of blood–milk barrier crossing by Zika Virus (ZIKV). In order to explain the presence of ZIKV particles in breast milk of infected mothers, several mechanisms could occur. Free viral particles (dark red) present in the bloodstream could sequentially infect myoepithelial cells (orange) and luminal cells (pink), resulting in the production of free viral particles in breast milk. Infected immune cells from the bloodstream (blue) could cross the epithelial barrier via the paracellular route by the « Trojan horse » mechanism, resulting in the presence of infected cells in breast milk that could produce viral particles. The viral particles produced could productively infect the luminal cells of the epithelium resulting in the presence of both cell-free and cell-associated ZIKV in breast milk. Infected epithelial cells could also be found in breast milk after being split off from the epithelium. Finally, it cannot be excluded that vesicles containing multiple virions could be produced by the mammary epithelium.

Taken together, these results suggest a role of the mammary epithelium in the production and excretion of viral particles into breast milk, but we cannot exclude that circulating immune cells infected by ZIKV (e.g., monocytes, lymphocytes, etc.) could contribute to the entry of ZIKV into the lactiferous compartment during their transmigration from the bloodstream to breast milk, with the so-called “Trojan horse” mechanism. However, persistence of viral RNA in breast milk suggests the existence of a reservoir within the mammary gland. Infected immune cells could transmigrate through the mammary epithelium during the viremic state, then newly produced particles could infect luminal epithelial cells in the alveolar compartment, serving as a reservoir in the mammary gland as long as the lactation state is maintained (Figure 3). As the nature of infectious viral entities (free, cell-associated, or vesicle-cloaked particles) in breast milk is unknown, these three mechanisms are plausible to this day.

**Step 2. Effect of human breast milk on viral infectivity.**

**Breast milk composition**—Breast milk is a biological fluid synthesized by the mammary glands of all female mammals after delivery. Biochemically, whole breast milk is defined as an emulsion of fatty globules rich in triglycerides (cream) and immersed in a plasma solution. This plasma solution, or skim milk, is a suspension of insoluble casein micelles bathed in the lactoserum, also called whey. The lactoserum is particularly rich in carbohydrates (lactose), proteins (α-lactalbumin, lactoferrin, lysozyme, Igs, etc.), mineral salts, and vitamins. Breast milk contains maternal immune cells (mainly myeloid precursors, neutrophils, immature granulocytes, and T lymphocytes) [45] and maternal breast epithelial cells.

Many studies have reported the benefits of breastfeeding, such as protection against respiratory infections, obesity, and sudden infant death syndrome [46]. However, although breastfeeding provides many benefits to the infant, it may also not be recommended in some cases of viral infection of the mother, as breast milk is a source of transmission of several viruses [12]. These viruses can be transmitted as free viral particles like HIV-1 [47] and CMV [48], or in a cell-associated form, such as HIV-1 [47] and HTLV-1 [39]. Other viruses could be transmitted through extracellular vesicles, since epithelial cells were shown to be able to produce vesicle-cloaked viral clusters of rotavirus (RV) or norovirus (NV). These vesicle-cloaked viral clusters provide an increase in viral stability and multiplicity of infection [49].

The simultaneous presence of breast milk components and viral entities could lead to interactions inducing an enhanced or decreased viral transmission to the newborn through breastfeeding.

**Effect of breast milk on the three main milk-borne viruses**—The effect of human breast milk on viral infectivity has been investigated in several studies, on the three main milk-borne viruses: HIV-1, CMV, and HTLV-1.

**Figure 4:**
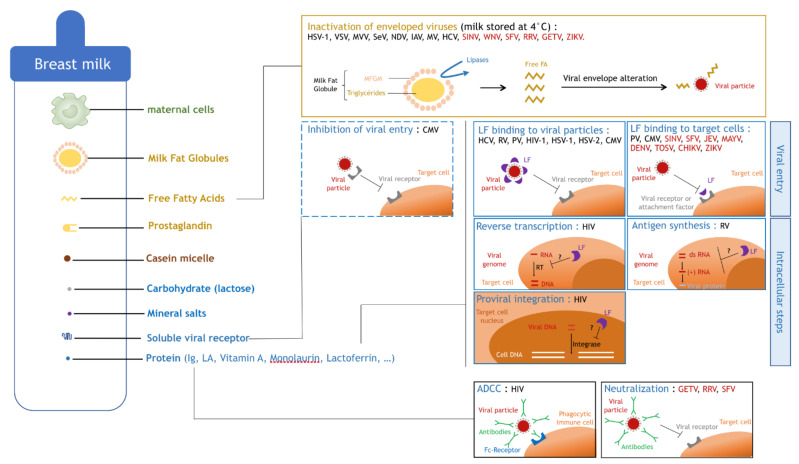
Inhibitory mechanisms of viral infectivity by breast milk components. Human breast milk contains components of different natures such as maternal cells (in green), the cream fraction (in yellow), the skim milk fraction composed of lactoserum (in blue) and casein micelles (in brown) (Figure 4). The cream fraction contains milk fat globules, free fatty acids (free FAs), and prosta-glandins. The lactoserum contains carbohydrates such as lactose, mineral salts, and a high diversity of proteins such as immunoglobulins (Ig), lactalbumin (LA), vitamin A, monolaurin, lactoferrin (LF), soluble viral receptors, and others. Lipases contained in breast milk digest triglyceride hearts of MFG when the milk fat globule membrane (MFGM) is altered, generating free FA (yellow box). Free FA inactivate enveloped viruses such as Herpes simplex virus type 1 (HSV-1), Vesicular stomatitis virus (VSV), Maëdi–Visna virus (MVV), Sendaï virus (SeV), Newcastle disease virus (NDV), type A Influenza virus (IAV), Measles virus (MV), Hepatitis C virus (HCV), and arboviruses (red) such as Sindbis virus (SINV), West Nile virus (WNV), Semliki forest virus (SFV), Ross river virus (RRV), Getah virus (GETV), and Zika virus (ZIKV) by interacting with the viral envelope and altering viral particle integrity (yellow box). Soluble viral receptors present in breast milk inhibit viral entry of viruses such as Cytomegalovirus (CMV) by interacting with viral par-ticles contained in breast milk, thus inhibiting viral binding to cell receptors on target cells (blue box, dashed line). LF inhibits viral entry by binding either to viral particles or to target cells (blue boxes, full line, first row). LF binds to HCV, Rotavirus (RV), Poliovirus (PV), type 1 Human immunodeficiency virus (HIV-1), HSV-1, Herpes simplex virus type 2 (HSV-2), or CMV. LF binds to target cells, inhibiting infection by PV, CMV, and arboviruses (red) such as SINV, SFV, Japanese encephalitis virus (JEV), Mayaro virus (MAYV), dengue virus (DENV), Toscana virus (TOSV), Chikungunya virus (CHIKV), or ZIKV. LF supposedly inhibits intracellular steps of the viral cycle like reverse transcription (RT), antigen synthesis, or proviral integration into the host cell genome by yet unknown mechanisms (blue boxes, full line, second and third rows). Maternal Igs present in breast milk can bind to viral particles, thus inhibiting their infectivity either by antibody-dependent cell-mediated cytotoxicity (ADCC) or by neutralizing (black boxes, full line) arboviruses (red) such as GETV, RRV, and SFV.

It was demonstrated, in vivo and in vitro, that human breast milk induces a decrease in HIV-1 infectivity. Pre-incubation of HIV-1 with human breast milk leads to a dose-dependent reduction of HIV-1 infectivity in vitro, and a reduction of oral transmission to mice in vivo [50]. An antiviral mechanism was suggested by Mabuka et al., as they showed that HIV-specific antibodies capable of antibody-dependent cell-mediated cytotoxicity (ADCC) are common in breast milk and are associated with a reduced risk of transmission in women with high viral loads [51]. Another breast milk component, the lactoferrin (LF), was shown to interfere with HIV-1 infection [52,53], probably by inhibiting viral adsorption (Figure 4).

Concerning the transmission of CMV during breastfeeding, it was shown that different breast milk components have opposite effects on viral transmission. Clarke et al. showed that vitamin A, monolaurin, and LF have an antiviral effect, while prostaglandins PGE2 and PGF2α promote viral replication [48]. They also found that soluble forms of viral receptors compete with cellular receptors for binding of CMV, leading to a reduced adsorption of viral particles to the target cell membrane (Figure 4).

Finally, it has been largely determined that breastfeeding over 6 months is associated with MTCT of HTLV-1 [54]. The effect of breast milk or its components on HTLV-1 infectivity has not yet been studied but, as component concentrations vary over time during breastfeeding, it is quite plausible that some of them may have an effect on HTLV-1 transmission.

**Effect of lactoferrin on viral infectivity**—The decrease in viral infectivity has often been associated with the presence of LF (mentioned above), an iron-binding protein present in breast milk. Indeed, it has been widely accepted since the 1990s that LF inhibits viral replication by interfering with viral adsorption at the cell membrane (Hepatitis C virus (HCV) [55,56], RV [57], Poliovirus (PV) [58], HIV-1 [52,53,59], Herpes simplex virus type 1 (HSV-1) [60,61,62,63,64], Herpes simplex virus type 2 (HSV-2) [60,62], and CMV [48,53,65,66]) (Figure 4). Several mechanisms have been described at different steps of the viral cycle. LF most probably inhibits the viral entry by binding either to viral particles or to target cells, thus preventing their interaction. Indeed, it was demonstrated that LF is capable of binding to viral envelope proteins such as E1 and E2 of HCV [67] or gp120 of HIV-1 [68], as well as heparins of the target cell membrane during CMV infection [69,70,71,72]. Other teams also found that LF can act during later steps of the viral cycle. Superti et al. suggested that LF not only interferes with viral entry but also with viral antigen synthesis during active RV infection, maintaining an antiviral effect after entry [57]. Finally, Ng et al. suggested that LF could inhibit HIV-1 reverse transcriptase in a dose-dependent manner, and weakly inhibits HIV-1 protease and integrase [73] (Figure 4).

On the other hand, LF has also been shown to exert a proviral effect on HTLV-1 replication. LF upregulates HTLV-1 LTR promoter activity resulting in an enhanced viral expression [59]. Moriuchi et al. also found that the viral protein Tax transactivates the LF gene promoter in myeloid-differentiated cells (HL-60) or mammary epithelial cells (MCF-7), inducing the expression of LF and thus facilitating milk-borne transmission of HTLV-1 [74].

The effect of LF on arboviruses is detailed in a further paragraph.

**Effect of free fatty acids on viral infectivity**—The reduction of viral infectivity can also be associated with an antiviral activity of lipids in breast milk. Welsh et al. showed that unsaturated fatty acids and monoglycerides present in breast milk inactivate enveloped viruses but not non-enveloped ones [75], suggesting that breast milk lipids disorganize the viral envelope. Thormar et al. confirmed these results but also found that free fatty acids become antiviral after breast milk storage at 4 °C and in infants’ stomachs within 1 h of feeding [76]. It was shown by Pfaender et al. that after storage at 4 °C, concentrations of free fatty acids in breast milk increase, which could explain the antiviral effect observed. They suggested that, upon freezing and thawing, milk fat globule membranes surrounding milk fat globules (MFGs) become altered and triglycerides inside the MFGs become accessible to lipases present in breast milk, resulting in an increase of free fatty acids [77,78]. The pre-incubation of enveloped viruses with free fatty acids impairs the integrity of the viral envelope [76,79], confirming their antiviral role. The enveloped viruses tested were Vesicular stomatitis virus (VSV), HSV-1, Maëdi–Visna virus (MVV) [76], Sendaï virus (SeV), Newcastle disease virus (NDV), type A influenza virus (IAV), SINV and WNV [79]. Another observation confirming this model was made by Thormar et al. who observed that the skim fraction is needed for the lipids to become antiviral, suggesting that the lipases present in the skim fraction alter MFG integrity in the cream fraction [76] (Figure 4).

The effect of free fatty acids on arboviruses is discussed in the next paragraph.

**Effect of breast milk on arboviruses**—The effect of human breast milk on arboviral infectivity was first studied in 1977 by Welsh et al., who showed that breast milk has an antiviral activity on the replication of Getah virus (GETV), RRV, Semliki forest virus (SFV), SINV and Bebaru virus (BEBV). They identified the origin of the antiviral activity as being IgA neutralization for GETV, RRV, and SFV (but none was observed for SINV or BEBV) and the cream fraction for SFV [75] (Figure 4). Then, in 1980, Kohn et al. identified the free fatty acids as being responsible for the inhibition of SINV and WNV infectivity [79]. Free fatty acids also interact with HCV, as found by Pfaender et al. Although HCV is not an arbovirus, it is part of the *Flaviviridae* family and presents many similarities with arboviruses belonging to the *Flavivirus* genus, such as ZIKV, YFV, and DENV. It was shown that the storage of breast milk at 4 °C was responsible for both anti-HCV [77] and anti-ZIKV [80] activities. Finally, it was recently shown that the cream fraction is responsible for anti-ZIKV activity of human breast milk stored at −20 °C, +4 °C, +22 °C, or +37 °C by altering viral particle integrity [81].

In addition, LF was shown to have an antiviral effect on mosquito-borne viruses such as SINV [82], SFV [82], Japanese encephalitis virus (JEV) [83], Mayaro virus (MAYV) [84], DENV [85], Toscana virus (TOSV) [86], CHIKV [87], and ZIKV [87]. LF mostly interferes with viral entry by interacting with heparan sulfate proteoglycans (HSPGs) that are bound by flaviviruses before the receptor-mediated entry into the cell [88] (Figure 4).

Finally, it was recently suggested that components of the whey fraction can also be antiviral. Extracellular vesicles and glycosaminoglycans contained in breast milk prevent viral attachment to the cell surface of ZIKV and Usutu virus (USUV) [89].

It is essential to investigate the effect of breast milk on arbovirus stability and infectivity as high viral loads are present and persist in breast milk of infected mothers, thus representing a risk of transmission to the newborn and development of severe symptoms. Although the effect of breast milk has been studied on arboviruses such as GETV, RRV, SFV, SINV, WNV, BEBV, and recently, ZIKV, no data are yet available concerning other arboviruses that are probably transmitted during breastfeeding, such as YFV [33,34], DENV [90], and CHIKV [15].

**Milk treatment strategies to inactivate milk-borne viruses**—Different strategies, based on heating or freezing milk, have been tested to find efficient ways to neutralize viral infectivity in breast milk samples (Table 2) and allow women of any country to provide their infants with the benefits of breastfeeding, despite their infection. Eradication methods of viruses from breast milk that would not impair the structure or the amount of immunological and nutritional factors in breast milk would be a crucial tool to protect newborns from chronic infection.

The effect of long or rapid heating of milk samples containing infectious particles from CMV-positive mothers has been assessed. The Holder pasteurization process consists of heating at 62.5 °C for 30 min and was shown to effectively inactivate CMV particles in breast milk [91,92]. High-temperature short time (HTST) treatment consists of heating the milk samples at 72 °C for a few seconds and was also shown to efficiently neutralize CMV particles contained in breast milk [93]. Finally, freezing milk samples at −20 °C provided unclear results concerning the risk of transmission of CMV through breastfeeding. Although it was observed that milk storage at −20 °C for 3 days decreases 99% of viral infectivity [91], several research teams observed contradictory effects. Either the infectivity was not completely eliminated [94], or it was not eliminated in all the samples tested [95]. Therefore, CMV particles were not efficiently eliminated from breast milk stored at −20 °C, regardless of the length of storage from 4 to 10 days [94,95,96]. When the length of freezing was extended to 20 days, viral particles were completely inactivated in the milk samples [96]. The differences observed between these studies could depend on the viral load in the different breast milk samples. Among these strategies, only HTST treatment and freezing at −20 °C do not significantly impair breast milk composition in immunological and nutritional components [93,97].

The effect of different breast milk heating strategies on HIV-1 infectivity has been studied. The Holder pasteurization process was proven effective at viral inactivation in breast milk [98] but it leads to a decrease of IgA [99], vitamins [100], and LF [101]. Pretoria pasteurization consists of bringing water to a boil and immediately placing a jar of milk inside (while having removed the water from the heat source). This technique heats the milk to about 56 to 62.5 °C for 10 to 15 min. This technique is effective at viral inactivation [102] and does not significantly impair the immunological factor content of breast milk [103]. Flash-heat treatment consists of placing a jar of milk in water, then bringing it to a boil. This process was shown to efficiently inactivate HIV-1 particles in breast milk [103,104] without decreasing immunological components [105]. Although some of these treatments are effective and feasible at home, the WHO does not recommend any of these heat strategies but favors that HIV-positive mothers keep breastfeeding their infants while receiving highly active antiretroviral therapy (HAART) [106].

The effect of milk treatment strategies on HTLV-1 infectivity has unfortunately been understudied. To our knowledge, only freezing at −20 °C has been studied. This technique destroys the HTLV-1-infected cell content in breast milk [107]. In endemic regions such as Japan, pregnant women are screened for HTLV-1 antibodies [108]. When a mother is identified as infected, bottle feeding is recommended. Although this strategy was proven to be effective at decreasing MTCT of HTLV-1 during breastfeeding [109], it deprives newborns of the benefits of breastfeeding. Thus, milk treatment strategies would enable infected mothers to keep breastfeeding.

Very few studies have investigated the effect of milk treatment strategies on arboviruses. To our knowledge, the effect of different milk storage temperatures on viral infectivity has only been studied on ZIKV. As mentioned previously, breast milk storage at –20 °C, +4 °C, +22 °C, or +37 °C [81] and breast milk pasteurization [80] successfully inactivate viral particles. Although these studies provide valuable information, viral inactivation was observed using breast milk from healthy mothers after incubating free viral particles. In breast milk of infected mothers, however, the virus could be cell-free, cell-associated, or even vesicle-cloaked, possibly making these milk treatments inefficient against ZIKV in vivo.

**Step 3. Transmission to the newborn via oral route.**

After ingestion of breast milk, milk-borne viruses need to cross an epithelial barrier in order to disseminate into the host organism. They can cross the tonsillar and intestinal barriers, which are located on the way of infected breast milk in the digestive tract, or they could even cross the respiratory barrier as many newborns are affected by dysphagia. Dysphagia is characterized by a difficulty in swallowing and can result in the penetration of breast milk into the lungs. Although it is difficult to evaluate the incidence of dysphagia among infants, it was reported that it may affect up to 25% of all children [110].

### 3.1. Viral Crossing of the Tonsillar Barrier

**The tonsillar barrier**—Tonsils are lymphoid organs located in both the nasal and buccal cavities and play an important role in the immune response against respiratory and orally transmitted microorganisms.

In the buccal cavity, the tonsillar barrier is a pluristratified squamous epithelium that forms deep tonsillar crypts, at the base of which are found micropore cells that are specialized in antigen transport. Beneath the epithelium are located lymphatic nodules with germinal centers that participate in the immune function of the tonsils by producing new lymphocytes and activating the immune response [111]. Although tonsils allow recognition and elimination of foreign organisms, some viruses are able to cross them, either by infection or by transcytosis.

**Tonsillar barrier crossing by orally transmitted viruses**—Tonsillar barrier crossing has been determined for Epstein–Barr virus (EBV) and HIV.

It has been well documented that EBV particles are secreted into patients’ saliva [112,113,114,115] at high viral loads [113]. Two mechanisms of oral transmission were found. First, Tugizov et al. showed that EBV can cross an in vitro model of the tonsillar barrier through both apical-to-basolateral (AP-to-BL) and basolateral-to-apical (BL-to-AP) transcytosis, with approximative efficiencies of 10 and 20%, respectively. AP-to-BL transcytosis of EBV is initiated by macropinocytosis while caveolin-dependent endocytosis mediates BL-to-AP transcytosis [116]. Secondly, it was found that EBV infects the epithelial barrier, using primary tonsillar epithelial cells [117] or a stratified epithelium in vitro [118], resulting in a release of infectious viral particles by the BL pole.

HIV, a milk-borne virus, targets different cell types in the tonsils. In the late 1990s, HIV was shown to infect tonsillar leucocytes either on infected patients [119,120] or when exposed in vitro to cell-free HIV [121]. Another study identified HIV-infected immune cells in mechanically disaggregated human tonsillar tissue after in vitro infection as expressing CD4 (T cells), CD11c (dendritic cells), or CD68 (monocytes) [122]. However, in order to reach these immune target cells in vivo, the virus would have to cross the external epithelium. No mechanism has yet been determined but Maher et al. showed that HIV particles are capable of binding to external epithelial cells [123]. Although the mechanism of HIV crossing is not known, it was shown using simian models that cell-free HIV infects external epithelial cells within the tonsils of infected macaques [124], suggesting that the virus could reach the germinal centers by productively infecting the epithelial barrier. As viral entities of HIV from breast milk can be either cell-free or cell-associated [47], several mechanisms could co-exist to enable HIV crossing through the tonsillar barrier.

### 3.2. Viral Crossing of the Respiratory Barrier

In the first instance, it might seem peculiar to address interactions between milk-borne viruses and the respiratory barrier. However, the respiratory barrier crossing by milk-borne viruses could be relevant in the context of MTCT of arboviruses during breastfeeding as newborns are often affected by dysphagia, a process implicating breast milk penetration into the respiratory tract during drinking [110]. As breast milk of arbovirus-infected mothers contains high viral loads and/or infectious particles, this biological fluid may thus represent a risk of transmission to the newborn via the respiratory tract during breastfeeding.

The respiratory system is composed of the upper and lower respiratory tracts. The upper part refers to the nasal cavity, pharynx, and larynx. The lower part refers to the trachea, bronchi, bronchioles, and alveoli.

The respiratory epithelium varies in structure throughout the respiratory tract. Four different structures are found: the pseudo-stratified epithelium (in the nasal cavity and in the highest part of the lower tract), the stratified squamous epithelium that lines the pharynx, the simple cuboidal epithelium located in the bronchioles, and the simple squamous epithelium that allows gas exchanges in the alveoli [125]. Although different defense mechanisms co-exist along the respiratory tract, some viruses have acquired the ability to bypass them in order to infect the organism. Among them, rhinoviruses [126], coronaviruses [127,128], Respiratory Syncytial Virus (RSV) [129,130], and influenza viruses [131] alter the respiratory barrier integrity resulting in its disruption, while MERS-CoV [132] can also productively infect the respiratory epithelium and be released from its BL side.

Concerning arboviruses, ZIKV, JEV, WNV, and USUV were shown to productively infect primary nasal epithelial cells cultivated in air–liquid interface (ALI) conditions [133]. While no disruption of the barrier function was observed, only JEV and WNV were shown to efficiently be released basolaterally and cross the respiratory barrier. Those first data, in accordance with the detection of arboviral RNA in nasopharyngeal swabs, could also be a clue for potential infection of the newborn respiratory epithelium through exposure to contaminated breast milk.

### 3.3. Viral Crossing of the Intestinal Barrier

**Figure 5:**
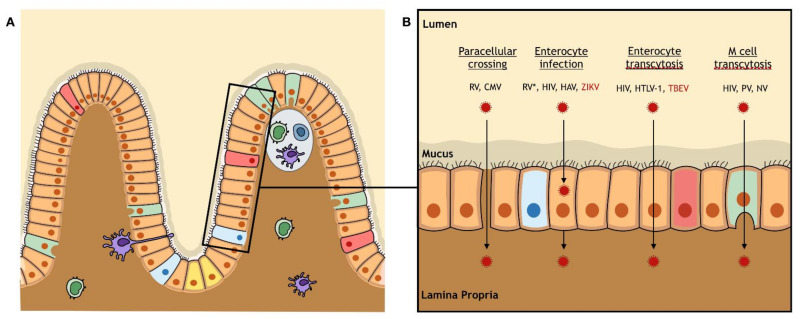
The intestinal barrier: anatomy and viral crossing. (**A**) The intestinal barrier is a monostratified epithelium composed of different cell types that all differentiate from pluripotent stem cells (yellow) located at the bottom of the crypts: enterocytes (orange), goblet cells (pink), Paneth cells (blue), and microfold cells (M cells) (green). Underlying the epithelium, the gut-associated lymphoid tissue is composed of free and anchored immune cells. Over the lymphoid follicles, the follicle-associated epithelium is enriched by M cells. (**B**) The intestinal barrier can be crossed by paracellular crossing by Rotavirus (RV), by enterocyte infection by RV (*: after paracellular crossing), Human immunodeficiency virus (HIV), Hepatitis A virus (HAV), and Zika virus (ZIKV), by transcytosis through enterocytes by HIV, type 1 Human T-cell lymphotropic virus (HTLV-1), and Tick-borne encephalitis virus (TBEV), or by transcytosis through M cells by HIV, Poliovirus (PV), and Norovirus (NV). The only arboviruses (red) that were reported to cross the intestinal barrier are TBEV and ZIKV.

**The intestinal barrier**—The intestinal barrier is a vast semi-permeable barrier that allows the absorption of nutrients while preventing the transport of pathogens. The intestinal barrier is a monostratified epithelium composed of different cell types. The most abundant cells are enterocytes, but other cells are found such as goblet cells, Paneth cells, and microfold cells (M cells) that all differentiate from intestinal stem cells located at the bottom of deep intestinal crypts (Figure 5). Under the epithelial monolayer is located the gut-associated lymphoid tissue (GALT), which is composed of isolated immune cells that are anchored to the monolayer or free in the lamina propria, lymphoid follicles such as Peyer patches and mesenteric lymph nodes. Overlying the follicles, the follicle-associated epithelium (FAE) is enriched by M cells that play a major role in the immune response and tolerance as they have a high capacity of transcytosis, thus allowing antigens crossing toward immune tissues. Although the intestinal epithelium is a tight monolayer expressing tight junction proteins that allow the barrier function, and containing the GALT that allows its immunologic function, some viruses have developed mechanisms to cross the intestinal barrier.

**Intestinal barrier crossing by orally transmitted viruses**—Several orally transmitted viruses have been found to cross the intestinal barrier: viruses transmitted through the oro-fecal route such as Hepatitis A virus (HAV), RV, PV, and NV, or viruses transmitted during breastfeeding such as HIV, CMV, and HTLV-1. A variety of mechanisms have been acquired by these viruses in order to cross the intestinal barrier (Figure 5). Some infect enterocytes without inducing a disruption of the monolayer, like HAV [134] or HIV [135], while others induce a loss of barrier integrity, like RV [136] and CMV [137]. Another viral crossing mechanism consists in transcytosis through enterocytes by viruses such as HIV [138] and HTLV-1 [139], or through M cells like HIV [135], PV [140], and NV [141] (Figure 5).

In the late 1980s, an in vitro model of an intestinal barrier was developed using the Caco-2 cell line (from a human colorectal adenocarcinoma). Caco-2 cells grown on polycarbonate filters for approximatively 20 days form a tight monolayer that allows studying viral crossing and barrier integrity. Using this model, the Caco-2 cell line was shown to be permissive to HAV [134], HIV [135], and RV [136]. The infection by HAV leads to a production of viral particles by both AP and BL sides with a lower production efficiency from the BL side and without loss of epithelium integrity. HIV infection of Caco-2 cells is dependent on the expression of a receptor called galactosyl-ceramide (GalCer) and leads to no disruption of the intestinal barrier. The infection of enterocytes by RV induces either an alteration of tight junctions or apoptosis in Caco-2 monolayers in vitro, and leads to a rupture of the intestinal barrier [136]. The apoptosis is induced through the mitochondrial pathway [142] and is dependent on the NSP4 viral protein. Interestingly, RV was shown to be produced in the form of large vesicles containing several virions in the feces of mice and pigs [49].

Other viruses cross the intestinal barrier by transcytosis through enterocytes. It was shown for HIV that the virus crosses a tight Caco-2 monolayer by transcytosis that is dependent on GalCer expression [138]. Bomsel et al. also showed that during BL-to-AP transcytosis, intracellular Igs target the viral particles, thus inhibiting the viral transport across the cell [143]. Transcytosis of HTLV-1 was demonstrated by our team using the in vitro model of Caco-2 tight monolayer [139]. We showed that transcytosis of HTLV-1 through enterocytes does not alter barrier integrity or the virus’s ability to infect underlying human dendritic cells.

Transcytosis of viral particles across the intestinal barrier was also shown to occur in M cells. It was shown on an in vitro model of Caco-2 monolayers enriched with M cells that HIV particles are transported across M cells by transcytosis. Briefly, to induce M cell differentiation, B cells were added to the BL side of Caco-2 monolayers and M cell differentiation was checked by microscopy and by measuring the transcytosis capacity of the monolayers. Only X4 virions were found to cross M cells by transcytosis [135]. PV was also shown to cross M cells by transcytosis [140]. It was found that after PV type 1 infection of human Peyer’s patches explants, viral particles would preferentially adhere to M cells, and intracellular vesicles containing virions were observed by electron microscopy. The penetration of PV into M cells was also confirmed in vivo on a simian model [144]. NV was shown to cross polarized murine intestinal epithelia in vitro by transcytosis through M cells [141]. These results were confirmed in vivo on a mouse model. After oral inoculation of NV, transient depletion of M cells was associated with a decreased infection [145]. Interestingly, it was also shown that NV is shed in feces in the form of vesicles containing numerous viral particles that remain intact during feco-oral transmission [49].

### 3.4. Viral Crossing of Epithelial Barriers by Arboviruses

After inoculation of infected breast milk through the oral tract, milk-borne arboviruses could cross the three epithelial barriers mentioned above: the tonsillar, the respiratory, and the intestinal barriers.

No arbovirus has yet been found to cross the tonsillar barrier, possibly due to the lack of research on that topic. The tonsillar epithelium could be an entry site for arboviruses as infected breast milk is in contact with the tonsils during its transit in the oral tract. The mechanisms described above for orally transmitted viruses could be used by milk-borne arboviruses to cross the tonsillar barrier.

Concerning the respiratory barrier crossing, several arboviruses (JEV and WNV) were shown to cross an in vitro model of the nasal epithelium, but no study was performed on primary human airway epithelia. Although the mechanisms described could be used by milk-borne arboviruses, viral dissemination into the host organism most likely occurs across the intestinal barrier.

In the context of breastfeeding, arboviruses are likely to reach the intestinal barrier as the pH of newborns’ stomachs is buffered by breast milk and significantly higher than the pH of an adult stomach [146]. Arboviruses like ZIKV [147] or TBEV [148] were shown to be stable at low pH or even in gastric juices for TBEV [149]. To this day, TBEV and ZIKV are the only arboviruses that have been shown to cross the intestinal barrier in experimental models. Using the Caco-2 model, Yu et al. showed that TBEV crosses the monolayers via transcytosis during the early stage of infection, and via the paracellular route five days after infection because of an impaired barrier integrity [150] (Figure 5). In our laboratory, we showed that the exposure of different Caco-2 clones to several ZIKV strains (isolated in Brazil in 2016 or in French Polynesia in 2013) leads to their infection. Using the polarized Caco-2 monolayer model, we observed no alteration of the epithelial barrier integrity and found a production of infectious viral particles from both the AP and BL sides, with a lower production efficiency from the BL side (Figure 5). In vivo, we demonstrated that oral inoculation of ZIKV to newborn A129 mice leads to their infection associated with neuro-invasion and a high mortality rate [151]. Additionally, we showed that the infection of breastfeeding dams post-partum results in a transmission of ZIKV RNA to their pups [151], thus demonstrating that breastfeeding might be a transmission route of ZIKV.

## 4. Discussion, Unanswered Questions and Future Directions

Both epidemiological and physiological evidence has been obtained regarding MTCT of arboviruses during breastfeeding. Viral genomes of CHIKV, DENV, WNV, and ZIKV were detected in mature breast milk or colostrum of infected women and shown to persist for long periods of time, even after viral clearance from the bloodstream (CHIKV and ZIKV). Additionally, infectious viral particles of DENV and ZIKV were detected in breast milk. Due to either limited volumes or low viral loads, the infectious nature of breast milk was not assessed for CHIKV, WNV, and YFV, but the existence of infectious particles in breast milk is not excluded. In several cases, newborns of arbovirus-infected mothers were also infected while no viral genome was detected from umbilical cord blood, eliminating the possibility of transplacental transmission. Although it is difficult to exclude a possible vector-borne transmission, two arguments demonstrate YFV transmission through breastfeeding and strongly suggest ZIKV transmission. First, three cases of transmission of the vaccine strain 17DD of YFV (17DD-YFV) occurred after post-partum vaccination of breastfeeding mothers, leading to the infection of their newborn with 17DD-YFV. Because the 17DD-YFV can infect the *Aedes aegypti* midgut but does not disseminate to the salivary glands, preventing its transmission to a novel host [152,153,154,155], the infection of the newborns with this vaccine strain demonstrates the transmission through breastfeeding. In parallel, WT YFV was shown to be excreted in breast milk, as vRNA was recently detected in mature milk [26]. Secondly, ZIKV was transmitted from a mother to her child and sequencing of breast milk and urine isolates revealed 99% of sequence identity. The advanced age of the child (5-month-old) excludes transplacental transmission and his very limited contact with the exterior, as well as the air-conditioning, limit a possible mosquito-borne transmission.

To explain MTCT during breastfeeding, three mechanisms are mandatory: viral dissemination to the mammary gland, viral stability in breast milk, and epithelial barrier crossing.

Due to the lack of studies on viral infection of the mammary glands, only ZIKV was shown, by our team, to disseminate to mammary glands after sub-cutaneous infection. Although we showed an important role of the mammary epithelium in viral replication and excretion into breast milk, other mechanisms cannot be excluded. As maternal immune cells cross the blood–milk barrier during lactation, infected immune cells could be present in breast milk. Immune cells in lactiferous ducts could also produce viral particles that could infect the epithelium, creating a viral reservoir that would explain the persistence of ZIKV in breast milk after its clearance from the bloodstream [17]. Finally, a desquamation of the mammary epithelium could lead to the presence of infected epithelial cells in breast milk. To determine which mechanism is responsible for breast milk infectivity, the form of the virus in breast milk should be identified using milk from infected mothers. As there is no ongoing epidemic, mice models are essential. Mouse milking was described in several publications [156,157,158] but the process is delicate and provides small volumes of breast milk, making it complicated to determine whether infected cells are present.

For breast milk to be infectious, viral stability needs to be maintained. Several studies have found an antiviral effect of breast milk on arboviruses. Since 1977, the cream fraction has been thought to be responsible for inactivating enveloped viruses in breast milk but recent data have challenged this assumption, as it was determined in several studies that the antiviral activity is only observed after milk treatment (4 °C or heat treatment). Fresh milk did not show any antiviral activity on ZIKV (or HCV) and still represents a risk in transmission of infectious particles to the newborn during breastfeeding.

LF was shown to inhibit the infection by many mosquito-borne viruses by interacting with HSPG, but all studies (except one [85]) were performed in vitro, in the absence of breast milk and its multiple components. One study was performed on mice models but the virus was pretreated with LF alone in vitro before being injected intra-cranially. The study of each breast milk component on its own is not relevant to the overall effect of breast milk on viral infectivity. For CMV and HIV-1, different breast milk components were shown to be antiviral on their own; however, both these viruses are still transmitted during breastfeeding. Therefore, two approaches should be prioritized to appropriately investigate the effect of breast milk on viral infectivity: the study of viral inactivation in vitro should be performed in the presence of fresh breast milk from either infected or non-infected mothers (as it was shown by Conzelmann et al. that preincubation of ZIKV with fresh breast milk does not result in viral inactivation), and in vivo studies should be carried out to study transmission and infection of breastfed pups. Animal models were used to demonstrate the transmission during breastfeeding of DENV [90], WNV [159], and SFV [159] using hamster pups, as well as ZIKV [151] and WNV [160] using mouse pups.

After oral inoculation, arboviruses present in breast milk need to cross an epithelial barrier. It could be either the tonsillar, intestinal, or respiratory barrier, but milk-borne arboviruses most likely cross the intestinal barrier. Today, the only arbovirus for which MTCT through breastfeeding has been studied, in an experimental murine model, is ZIKV. We showed, in vivo, that ZIKV is transmitted from infected mothers to breastfed newborn mice and we identified, in vitro, that the intestinal barrier allows viral replication and excretion of infectious virions from both AP and BL sides of polarized enterocyte-like cells. Although ZIKV crosses the intestinal barrier, it does not exclude a potential role of the other epithelial barriers as the ability of ZIKV to cross them has not been investigated. For other flaviviruses potentially transmitted during breastfeeding, such as DENV or YFV, the intestinal barrier could be an entry site as they share many similarities with ZIKV.

## 5. Conclusions

Although the main transmission route of arboviruses is vector-borne, other modes of transmission have been determined following large outbreaks that occurred in the last decades. Neglected for long, MTCT during breastfeeding was found for the vaccine strain of YFV, strongly highlighted for ZIKV, and suggested for CHIKV, DENV, and WNV. Additionally, a milk-borne transmission of TBEV to humans following the consumption of infected dairy products was also demonstrated, thus highlighting viral stability in milk. However, the only arbovirus for which MTCT during breastfeeding has been studied at this point is ZIKV, which disseminates to the mammary glands of infected mothers, is excreted into breast milk, and crosses the intestinal barrier, infecting newborn mice. Understanding the mechanisms of dissemination to the mammary gland, excretion into breast milk, and epithelial barrier crossing is essential to develop efficient tools to prevent the transmission of infectious viral particles to the newborn. Breast milk from infected mothers would allow us to determine the nature of viral entities, and more advanced culture tools such as organoids could bring new insight concerning the associated mechanisms. Finally, the study of the mechanisms associated with viral inactivation in breast milk could allow us to improve milk treatment strategies.

**Additionnal remark about the search strategy**: The search strategy was devised on the PubMed database for articles published up to 28 February 2021. Briefly, it included searches for the following items: $1 (“Arbovirus Infections” [Mesh]) OR (arbovirus OR arbovirus infections OR flavivirus OR togavirus OR bunyavirus OR reovirus); $2 (“Breast Feeding” [Mesh:NoExp] OR “Milk, Human” [Mesh]) OR (breast feeding OR breastmilk OR breast milk OR breast fed OR breastfed OR human milk OR lactation); the combination of these two search strategies produced 405 results. A second search strategy included a combination of $3 (“Arbovirus Infections” [Mesh]) OR (arbovirus OR arbovirus infections OR flavivirus OR togavirus OR bunyavirus OR reovirus)) AND ((“Breast Feeding” [Mesh:NoExp] OR “Milk, Human” [Mesh]) OR (breast feeding OR breastmilk OR breast milk OR breast fed OR breastfed OR human milk OR lactation)) with $4 ((“Disease Transmission, Infectious” [Mesh:NoExp]) OR “Infectious Disease Transmission, Vertical” [Mesh]) OR ((transmission OR detection OR persistence) AND (virus)) that led to 196 references. The search strategy was initiated by C. Cecilio and one author (S.D.) screened the titles and abstracts of articles identified from the database search. For potentially eligible articles, full-text papers were obtained and independently processed for data extraction by S.D., P.E.C and A.V. In very few cases, responding to such requirements, works found on Google Search were also included.

## Figures and Tables

**Table 1:** Epidemiological and clinical evidence of milk-borne arboviruses.

Virus	Genus	Evidence of MTCT during Breastfeeding	References
CHIKV	*Alphavirus*	Viral genome detected in breast milk 3 and 23 days after symptom onset. Persistence in breast milk after viral clearance from the blood stream.	[15]
DENV	*Flavivirus*	Viral genome detected in breast milk 1 to 14 days after symptom onset. Persistence of viral genome in breast milk. Infection of breastfed newborn but absence of viremia in umbilical cord blood.	[16]
		Viral genome and infectious viral particles in breast milk. Infection of breastfed newborns (while absence of viremia in umbilical cord blood of one of them).	[18]
WNV	*Flavivirus*	Viral genome detected in breast milk 16 days after transfusion.	[19]
		Viral genome detected in colostrum.	[20]
YFV	*Flavivirus*	Vaccination of breastfeeding mothers after delivery led to the presence of viral genome in their infant’s CSF.	[21,22]
		Vaccination of breastfeeding mothers after delivery led to the development of meningoencephalitis by her newborn.	[33,34]
		Viral genome of wild-type YFV detected in breast milk.	[26]
ZIKV	*Flavivirus*	Viral genome and infectious particles detected in breast milk. Persistence up to 33 days after symptom onset. Persistence after clearance from the blood stream. No transmission to the newborn but breastfeeding was avoided.	[17]
		Viral genome and infectious particles detected in breast milk. Transmission unclear (the only serum sample tested gave ambiguous results).	[25]
		Viral genome and infectious particles detected in breast milk. Transmission to the newborn (RT-qPCR+: serum and urine). Limited contact of the newborn with the exterior. Air-conditioned house. 99% of sequence identity between mother’s breast milk and infant’s urine isolates.	[23,24]
		Case 1: Viral genome but no infectious particles detected in breast milk (day 5 post-symptom onset). Transmission to the newborn (RT-qPCR+: serum and saliva). Case 2: Viral genome but no infectious particles detected in breast milk (day 5 post-symptom onset). Persistence after clearance from the blood stream. Transmission to the newborn (RT-qPCR+: serum and urine).	[27,35]
		Case 1: Viral genome and infectious particles detected in breast milk. No transmission to the newborn (RT-qPCR-: urine). Cases 2–3–4: No viral genome in breast milk.	[28]
		Viral genome detected in breast milk. Persistence? Mother presented symptoms at 9 weeks of gestation but viral genome detected 2 days post-delivery. Sequences of mother’s breast milk and infant’s urine clustered together.	[30]
		Viral genome detected in breast milk. Transmission to the newborn? (development of secondary microcephaly. But RT-qPCR-: serum).	[31]
		Viral genome detected in breast milk. No transmission to the newborn (RT-qPCR-: urine).	[32]

Abbreviations: Chikungunya virus (CHIKV), Dengue virus (DENV), West Nile virus (WNV), Yellow fever virus (YFV), Zika virus (ZIKV), Cerebrospinal fluid (CSF), Reverse transcription quantitative PCR (RT-qPCR).

**Table 2:** Viral inactivation strategies in breast milk.

Virus	Treatment	Viral Inactivation	Immunological (IgA, LF, Lysozyme) or Nutritional Components Composition	References
CMV	**Holder** **Pasteurization** (62.5 °C-30 min)	+	Decrease of immunological components	[91,97,100,101]
	**HTST** (72 °C-5–15 s)	+	No significant decrease of immunological components	[93]
	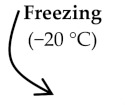	~	No significant decrease of immunological components	[97]
			**Viral inactivation**	**References**
		3 days	+	[91]
		4–10 days	+/−	[94]
		7 days	+/−	[95]
		10 days	-	[96]
		20 days	+	[96]
HIV-1	**Holder** **Pasteurization** (62.5 °C-30 min)	+	Decrease of immunological components	[97,98,99,100,101]
	**Pretoria** **pasteurization** (56–62.5 °C-10–15 min)	+	No significant decrease of immunological components	[102,103]
	**Flash-heat**	+	No significant decrease of immunological components	[103,104,105]
HTLV-1	**Freezing** (−20 °C–12 h)	+	ND	[107]
ZIKV	**+4 °C** (at least 1 day)	+	ND	[81]
	**+37 °C** (at least 2 h)	+	ND	[81]
	**−20 °C** (at least 10 h)	+	ND	[81]
	**+22 °C** (at least 10 h)	+	ND	[81]
	**Pasteurization** (62.5 °C-30 min)	+	ND	[80]

Legend: +: successful inactivation of viral infectivity. -: unsuccessful inactivation of viral infectivity. ~: unclear. +/−: not completely inactivated. ND: not determined.
